# Myocardial Injury and Predictors of In-Hospital Mortality in Severe or Critical COVID-19: A Single-Center Observational Cohort Study

**DOI:** 10.3390/medicina62091775

**Published:** 2026-09-16

**Authors:** Valentina Negrea, Septimiu Toader Voidazan, Adina Huțanu, Vlad Adrian Pop, Anca Meda Văsieșiu

**Affiliations:** 1 Doctoral School of Medicine and Pharmacy, George Emil Palade University of Medicine, Pharmacy, Science, and Technology of Targu Mures, 540142 Târgu Mureș, Romania; 2 1st Infectious Diseases Clinic, Mureș Clinical County Hospital, 540064 Târgu Mureș, Romania; adrian-vlad.pop@umfst.ro (V.A.P.); anca-meda.vasiesiu@umfst.ro (A.M.V.); 3 Department of Epidemiology, George Emil Palade University of Medicine, Pharmacy, Sciences and Technology, 540142 Târgu Mureș, Romania; septimiu.voidazan@umfst.ro; 4 Department of Laboratory Medicine, George Emil Palade University of Medicine, Pharmacy, Science, and Technology of Targu Mures, 540142 Târgu Mureș, Romania; adina.hutanu@umfst.ro; 5 Department of Infectious Diseases, George Emil Palade University of Medicine, Pharmacy, Science and Technology of Targu Mures, 540142 Târgu Mureș, Romania

**Keywords:** COVID-19, in-hospital mortality, severe form, prognostic factors

## Abstract

*Background and Objectives*: In-hospital mortality remains high among patients with severe or critical COVID-19. This study aimed to identify independent predictors of in-hospital mortality and assess the prognostic value of myocardial injury in this high-risk population. *Materials and Methods*: This single-center observational cohort study included 172 adults hospitalized with severe or critical COVID-19 across multiple pandemic waves. Mortality-associated factors were assessed using logistic regression and receiver operating characteristic curve analysis. Myocardial injury was defined as hs-cTnT > 14 ng/L, measured on days 3–5 after admission. *Results*: Of 172 patients, 60 died during hospitalization (34.9%). In the primary multivariable model, based on 126 complete cases and 37 deaths, age (aOR = 2.55 per 10-year increase; 95% CI, 1.55–4.22), pre-existing cardiovascular disease (aOR = 3.06; 95% CI, 1.03–9.07), LDH (aOR = 6.31 per doubling; 95% CI, 2.70–14.78), and CT pulmonary involvement (aOR = 1.34 per 10% increase; 95% CI, 1.01–1.80), were independently associated with in-hospital mortality. In a separate analysis of 121 patients with 35 deaths, myocardial injury remained independently associated with mortality after adjustment for age, cardiovascular disease, and pulmonary involvement (aOR = 5.19; 95% CI, 1.82–14.77). Among 131 patients with complete phenotype data, mortality was 6.7% (2/30) in patients with neither component, 19.6% (11/56) with isolated respiratory involvement, 50.0% (3/6) with isolated myocardial injury, and 56.4% (22/39) with both components. The primary multivariable model had an apparent AUC of 0.898 (95% CI, 0.828–0.968). *Conclusions*: Age, LDH, and myocardial injury were independently associated with in-hospital mortality, while the associations of cardiovascular disease and CT involvement were attenuated in the multiple-imputation sensitivity analysis. Combined assessment of clinical, pulmonary, and cardiac factors may improve risk stratification, but these findings require validation in independent cohorts.

## 1. Introduction

SARS-CoV-2 infection has a wide range of clinical presentations, from asymptomatic or mild disease to severe and critical illness with respiratory failure, systemic complications, and death. Despite advances in prevention and treatment, including vaccination and immunomodulatory therapies, in-hospital mortality remains high among patients with severe or critical COVID-19, particularly those with comorbidities and multiorgan dysfunction. Identifying factors associated with poor outcomes therefore remains important for risk assessment and clinical management [1].

Poor outcomes in COVID-19 result from a complex interaction between systemic inflammation, endothelial dysfunction, immunothrombosis, and multiorgan injury. Composite risk-stratification tools, such as the 4C Mortality Score, have been developed and externally validated to integrate multiple readily available clinical variables into a single prognostic instrument [2]. Critical COVID-19 shares several features with viral sepsis, including dysregulated inflammation, impaired microcirculation, and coagulation activation, which may contribute to disease progression and mortality [3,4]. These processes may be more pronounced in older patients and those with comorbidities, who often have a pre-existing proinflammatory and prothrombotic state. Changes in cytokine profiles associated with disease severity and survival further support the role of systemic inflammation, although no single mediator reflects the full biological complexity of the disease [1,5,6]. Similarly, inflammatory abnormalities and biochemical markers of organ injury have been associated with mortality. Elevated D-dimer levels have also been linked to poorer outcomes, reflecting the thromboinflammatory component of COVID-19, although D-dimer remains a nonspecific marker [7].

Despite the extensive literature on COVID-19 prognosis, reported mortality rates and prognostic factors have varied across different patient populations [8]. Although older age, cardiovascular comorbidities, elevated lactate dehydrogenase (LDH) levels, and myocardial injury have individually been associated with adverse outcomes in COVID-19, their independent and complementary prognostic value remains difficult to interpret given the heterogeneity of published cohorts, differences in disease severity, biomarker definitions, treatment strategies, and the pandemic periods studied. Consequently, the prognostic performance of these factors may vary across patient populations and clinical settings [6,9,10]. In this context, evaluating prognostic factors in local cohorts may therefore improve risk stratification and patient management.

The aim of this study was to identify independent predictors of in-hospital mortality among adults hospitalized with severe or critical COVID-19.

## 2. Methods

### 2.1. Study Design and Setting

We conducted a single-center observational cohort study of patients hospitalized with severe or critical COVID-19 in the Infectious Diseases I Clinic and/or the intensive care unit (ICU) of Mureș County Clinical Hospital. The study period extended from 11 November 2020 to 30 March 2022, covering several pandemic waves. The study was reported in accordance with the Strengthening the Reporting of Observational Studies in Epidemiology (STROBE) guidelines.

### 2.2. Study Population

The study included 172 eligible adult patients with SARS-CoV-2 infection confirmed by reverse transcription polymerase chain reaction (RT-PCR) of nasopharyngeal samples. COVID-19 diagnosis and severity were classified according to World Health Organization (WHO) criteria [11]. Severe COVID-19 was defined as pneumonia accompanied by at least one of the following: peripheral oxygen saturation < 90% on room air, respiratory rate > 30 breaths/min, or clinical signs of severe respiratory distress. Critical COVID-19 was defined by the presence of acute respiratory distress syndrome (ARDS), sepsis, septic shock, or other conditions requiring life-sustaining treatment, including invasive or non-invasive mechanical ventilation and/or vasopressor therapy. Patients were classified as having severe or critical COVID-19 at hospital admission according to these criteria and were followed until discharge or death. ICU admission alone was not used to define critical disease.

Inclusion criteria were age ≥ 18 years, severe or critical COVID-19 at hospital admission, availability of the clinical and laboratory data required for the analysis, and a known in-hospital outcome (discharge alive or death). Patients were excluded if they were <18 years old, had mild or moderate COVID-19, or were transferred to another healthcare facility before their in-hospital outcome could be determined.

### 2.3. Data Collection and Variables

Clinical data were collected from medical records and the hospital electronic information system using a standardized data collection form. Demographic, clinical, laboratory, imaging, treatment, and outcome data were recorded for each patient.

Demographic and baseline clinical variables included age, sex, body mass index (BMI) when available, COVID-19 vaccination status, time from symptom onset to hospital admission, time from symptom onset to ICU admission, and comorbidities, including hypertension, cardiovascular disease (ischemic heart disease, heart failure, atrial fibrillation/other arrhythmias, and significant valvular heart disease), diabetes mellitus, chronic kidney disease, chronic lung disease (asthma and chronic obstructive pulmonary disease (COPD)), malignancy, and immunosuppression. Comorbidity burden was assessed using the age-adjusted Charlson Comorbidity Index (ACCI), calculated for each patient based on age and comorbidities documented at admission according to the original Charlson methodology.

To assess potential differences across pandemic periods, patients were classified according to the predominant circulating SARS-CoV-2 variant at the time of hospital admission. Four epidemiological periods were defined: pre-Alpha (through 28 February 2021), Alpha (1 March–30 June 2021), Delta (1 July–31 December 2021), and Omicron (from 1 January 2022). Classification was based on reported periods of variant predominance in Romania and Europe [12] and was used solely as an epidemiological variable because individual genomic sequencing was not available.

Clinical data included symptoms, peripheral oxygen saturation, disease severity, and the type of respiratory support required, including oxygen therapy by face mask and non-invasive or invasive ventilation.

Laboratory variables included hematological, inflammatory, coagulation, renal, and liver function parameters. C-reactive protein (CRP), D-dimer, ferritin, neutrophil-to-lymphocyte ratio (NLR), creatinine, and LDH were specifically evaluated in the prognostic analysis. For the primary analysis, laboratory values obtained within the first 24 h of hospital admission were used. Maximum and minimum values recorded during hospitalization were analyzed separately as indicators of disease severity and progression and were not considered predictors available at admission. According to the study protocol, high-sensitivity C-reactive protein (hs-CRP) and high-sensitivity cardiac troponin T (hs-cTnT) were intended to be measured from the same blood sample collected 3–5 days after admission. Measurements were unavailable in some patients when sampling was not performed within the intended window or collected samples could not be processed. The specific reason for missingness was not systematically recorded for each patient. Because these measurements were obtained after the baseline assessment, they were analyzed separately as in-hospital biomarkers. hs-cTnT was measured by electrochemiluminescence immunoassay using the Elecsys Troponin T hs assay on a cobas e analyzer (Roche Diagnostics GmbH, Mannheim, Germany). Myocardial injury was defined as an hs-cTnT level > 14 ng/L, corresponding to the 99th-percentile upper reference limit for the assay.

Pulmonary involvement was assessed by chest computed tomography (CT). Recorded imaging findings included ground-glass opacities, pulmonary consolidations, estimated percentage of lung involvement, pulmonary embolism, pneumothorax, and pneumomediastinum.

The primary outcome was in-hospital mortality, defined as death during hospitalization for the COVID-19 episode under study. Patients discharged alive were classified as survivors, whereas patients transferred to other healthcare facilities before their outcome was known were excluded. Secondary objectives were to compare the clinical, laboratory, imaging, and in-hospital characteristics of survivors and non-survivors; evaluate the prognostic association between myocardial injury identified during hospitalization and mortality; explore cardiorespiratory phenotypes; and assess the apparent discriminatory performance of individual predictors and a multivariable model comprising a limited number of clinically relevant predictors available at hospital admission.

### 2.4. Statistical Analysis

Statistical analyses were performed using IBM SPSS Statistics, version 23 (IBM Corp., Armonk, NY, USA). Categorical variables were reported as frequencies and percentages. Continuous variables were reported as mean ± standard deviation (SD) or median and interquartile range (IQR), depending on their distribution. Normality was assessed using the Shapiro–Wilk test and graphical inspection.

Patient characteristics were compared between survivors and non-survivors. Continuous variables were compared using the independent-samples Student’s t-test for approximately normally distributed data and the Mann–Whitney U test for non-normally distributed data. Categorical variables were compared using Pearson’s χ^2^ test or Fisher’s exact test, as appropriate.

Differences in in-hospital mortality across pandemic periods (Pre-Alpha, Alpha, Delta, and Omicron) were assessed using Pearson’s chi-square test, with pandemic period treated as a categorical variable.

Potential predictors of in-hospital mortality were initially selected from variables available at hospital admission and included age, sex, hypertension, pre-existing cardiovascular disease, diabetes mellitus, malignancy, cerebrovascular disease, chronic obstructive pulmonary disease, chronic kidney disease, oxygen saturation (SaO_2_), extent of pulmonary involvement on CT, CRP, NLR, platelet count, LDH, creatinine, ferritin and D-dimer levels. Factors associated with in-hospital mortality were first assessed using univariable logistic regression. Variables associated with mortality at *p* < 0.05 were considered eligible for multivariable analysis. Variables reflecting subsequent disease progression, such as ICU transfer or critical COVID-19 during hospitalization, were not included as baseline predictors of mortality. Results were reported as odds ratios (ORs) with 95% confidence intervals (CIs). Because of their right-skewed distributions, CRP, NLR, LDH, creatinine, ferritin, D-dimer, and hs-cTnT were log_2_-transformed for logistic regression analyses; accordingly, the corresponding ORs represent the change in the odds of in-hospital mortality associated with a doubling of the respective biomarker concentration.

A primary multivariable logistic regression model was constructed to identify factors independently associated with in-hospital mortality using variables available at or shortly after hospital admission. Given the number of outcome events, a parsimonious set of clinically and statistically relevant, non-redundant predictors was selected from the variables eligible after univariable analysis, taking into account data availability and potential collinearity. The final model included four predictors representing three prognostic domains of interest: baseline vulnerability (age and pre-existing cardiovascular disease), systemic disease severity (LDH), and the extent of pulmonary involvement on CT. Selected predictors were entered simultaneously into the multivariable logistic regression model using the Enter method. No stepwise variable-selection procedure was applied. Results were reported as adjusted odds ratios (aORs) with 95% CIs and *p* values. The primary multivariable logistic regression model was internally validated using 2000 bootstrap resamples, with estimation of the optimism-corrected AUC and assessment of calibration using the calibration intercept and slope. Because the preceding candidate-screening process was not repeated within each bootstrap sample, the optimism correction pertains to the fixed final model rather than to the entire model-building procedure. The primary multivariable analysis was based on complete cases. As a sensitivity analysis, missing predictor data were handled using multiple imputation by chained equations with 50 imputations, including the outcome and primary-model predictors, and estimates were pooled using Rubin’s rules.

To account for potential temporal confounding across successive pandemic waves, a sensitivity analysis was performed by additionally adjusting the primary multivariable model for pandemic period, entered as a categorical variable representing the periods of predominance of the pre-Alpha, Alpha, Delta, and Omicron variants.

Multicollinearity among variables included in the multivariable logistic regression models was assessed using variance inflation factors (VIFs), with VIF values < 5 considered indicative of the absence of relevant multicollinearity.

Because hs-cTnT was measured on days 3–5 rather than at baseline, myocardial injury was evaluated in a separate targeted analysis and was not included in the primary multivariable model. A second multivariable logistic regression model was therefore used to assess whether myocardial injury, defined as hs-cTnT > 14 ng/L, was associated with in-hospital mortality after adjustment for age, pre-existing cardiovascular disease, and the extent of pulmonary involvement on chest CT, selected as clinically relevant potential confounders of the association between myocardial injury and mortality. As a sensitivity analysis, myocardial injury was alternatively defined using the sex-specific 99th-percentile hs-cTnT thresholds for the Roche Elecsys assay (>9.0 ng/L in women and >16.8 ng/L in men).

For the analysis of cardiorespiratory involvement, patients were classified according to the presence of myocardial injury and severe respiratory involvement, defined as SpO_2_ < 90% at admission and/or pulmonary involvement of ≥50% on CT. In-hospital mortality was compared across four phenotypes: no cardiac or severe respiratory involvement, isolated respiratory involvement, isolated cardiac involvement, and combined cardiorespiratory involvement. Because of sparse cells, the comparison between the combined cardiorespiratory phenotype and the reference phenotype was reassessed using Firth penalized logistic regression.

The discriminatory ability of the main mortality predictors was assessed using receiver operating characteristic (ROC) curve analysis. The area under the ROC curve (AUC) and 95% CI were calculated for each predictor. Discrimination of the final multivariable model was also assessed using the AUC.

Except for the multiple-imputation sensitivity analysis, analyses were performed using available cases for each variable without imputation of missing data. All tests were two-sided, and a *p* value < 0.05 was considered statistically significant.

### 2.5. Ethical Considerations

Three patients hospitalized before formal ethical approval were included after providing written informed consent; their pre-approval clinical data were obtained exclusively from routine-care medical records, and no study-specific day 3–5 blood samples were collected. Protocol-driven sampling was performed only after ethical approval. Patient data were anonymized before analysis, and access to the study database was restricted to members of the research team.

## 3. Results

A total of 172 adults with severe or critical COVID-19 were included between 11 November 2020 and 30 March 2022. Of these, 112 (65.1%) survived and 60 (34.9%) died during hospitalization. Baseline demographic, clinical, and laboratory characteristics are presented in Table 1. The study cohort and analytic samples used in the main and secondary analyses are summarized in Supplementary Figure S1.

Patients were admitted across all four epidemiological periods corresponding to the predominant circulating SARS-CoV-2 variants. In-hospital mortality differed significantly across pandemic periods (Pearson’s χ^2^ = 26.56, *p* < 0.001), with rates of 20.8% in the Pre-Alpha period, 25.0% during Alpha, 69.6% during Delta, and 60.0% during Omicron. Treatment patterns according to pandemic period and in-hospital outcome are summarized in Supplementary Table S1. Only five patients were vaccinated, of whom four died; vaccination was confined to the Alpha (n = 1), Delta (n = 2), and Omicron (n = 2) periods. Non-survivors were significantly older than survivors and had higher rates cardiovascular disease and chronic kidney disease; hypertension was also more frequent among non-survivors (*p* = 0.049). At admission, non-survivors had lower oxygen saturation, greater pulmonary involvement on CT, and significantly higher NLR, LDH, and D-dimer levels. hs-cTnT, measured on days 3–5 of hospitalization, was also significantly higher among non-survivors. The maximum CK level recorded during hospitalization was also significantly higher among non-survivors. Non-survivors also had higher AST levels than survivors (*p* = 0.002). CRP, platelet count, creatinine, ferritin, and ALT did not differ significantly between groups.

Overall comorbidity burden, assessed using the ACCI, was significantly higher among non-survivors. The median ACCI was 3 (IQR, 1.75–5) in the overall cohort, 4 (IQR, 3–6) among non-survivors, and 3 (IQR, 1–4) among survivors (*p* < 0.001).

In univariable logistic regression analysis of the evaluated variables, age, cardiovascular disease, chronic kidney disease, lower SpO_2_ at admission, greater pulmonary involvement on CT, NLR, D-dimer, hs-cTnT and LDH were significantly associated with in-hospital mortality. Each doubling of LDH was associated with a 3.99-fold increase in the odds of death (OR = 3.99; 95% CI, 2.14–7.42; *p* < 0.001). Among the in-hospital biomarkers measured on days 3–5, each doubling of hs-cTnT was associated with a 2.13-fold increase in the odds of death (OR = 2.13; 95% CI, 1.50–3.03; *p* < 0.001). In contrast, sex, diabetes mellitus, chronic lung disease, creatinine level, ferritin level, and platelet count were not significantly associated with mortality (Table 2).

The primary multivariable model included age, cardiovascular disease, extent of pulmonary involvement on CT, and LDH (Table 3). The analysis included 126 patients with complete data, of whom 37 died. Among the 46 patients excluded from the primary complete-case model, missing data included CT involvement in 24 patients, LDH in 26, and pre-existing cardiovascular disease in 2, with overlap between variables; 23 of these 46 patients died. No relevant multicollinearity was identified in the primary multivariable model (VIF range, 1.09–1.19). After adjustment, age, cardiovascular disease, the extent of pulmonary involvement on CT and LDH remained independently associated with in-hospital mortality. Each doubling of LDH was independently associated with a 6.31-fold increase in the odds of in-hospital mortality (aOR = 6.31; 95% CI, 2.70–14.78; *p* < 0.001). Within the same complete-case sample, the unadjusted OR for LDH was 4.46 (95% CI, 2.20–9.05; *p* < 0.001) per doubling. The apparent AUC was 0.898, while the optimism-corrected AUC after 2000 bootstrap resamples was 0.886, the optimism-corrected calibration intercept and slope were −0.036 and 0.905, respectively. The primary complete-case analysis included 126 of 172 patients (73.3%). In a multiple-imputation sensitivity analysis, the associations of age and LDH with in-hospital mortality remained robust, whereas the estimates for cardiovascular disease and CT involvement were attenuated (Supplementary Table S2).

In a sensitivity analysis using the same 126 complete cases (37 deaths) and additionally adjusted for pandemic period, age (aOR = 2.82 per 10-year increase, 95% CI, 1.48–5.38; *p* = 0.002) and LDH (aOR = 10.85 per doubling, 95% CI, 3.61–32.64; *p* < 0.001) remained independently associated with in-hospital mortality. The association between pre-existing cardiovascular disease and in-hospital mortality was attenuated and no longer statistically significant (aOR = 2.77, 95% CI, 0.80–9.61; *p* = 0.108), whereas the extent of pulmonary involvement on chest CT was not independently associated with mortality (aOR = 1.20 per 10% increase, 95% CI; 0.91–1.60; *p* = 0.194). However, this model was considered unstable because only 37 deaths were available for seven model parameters, and period-specific event counts were sparse.

**Figure 1:**
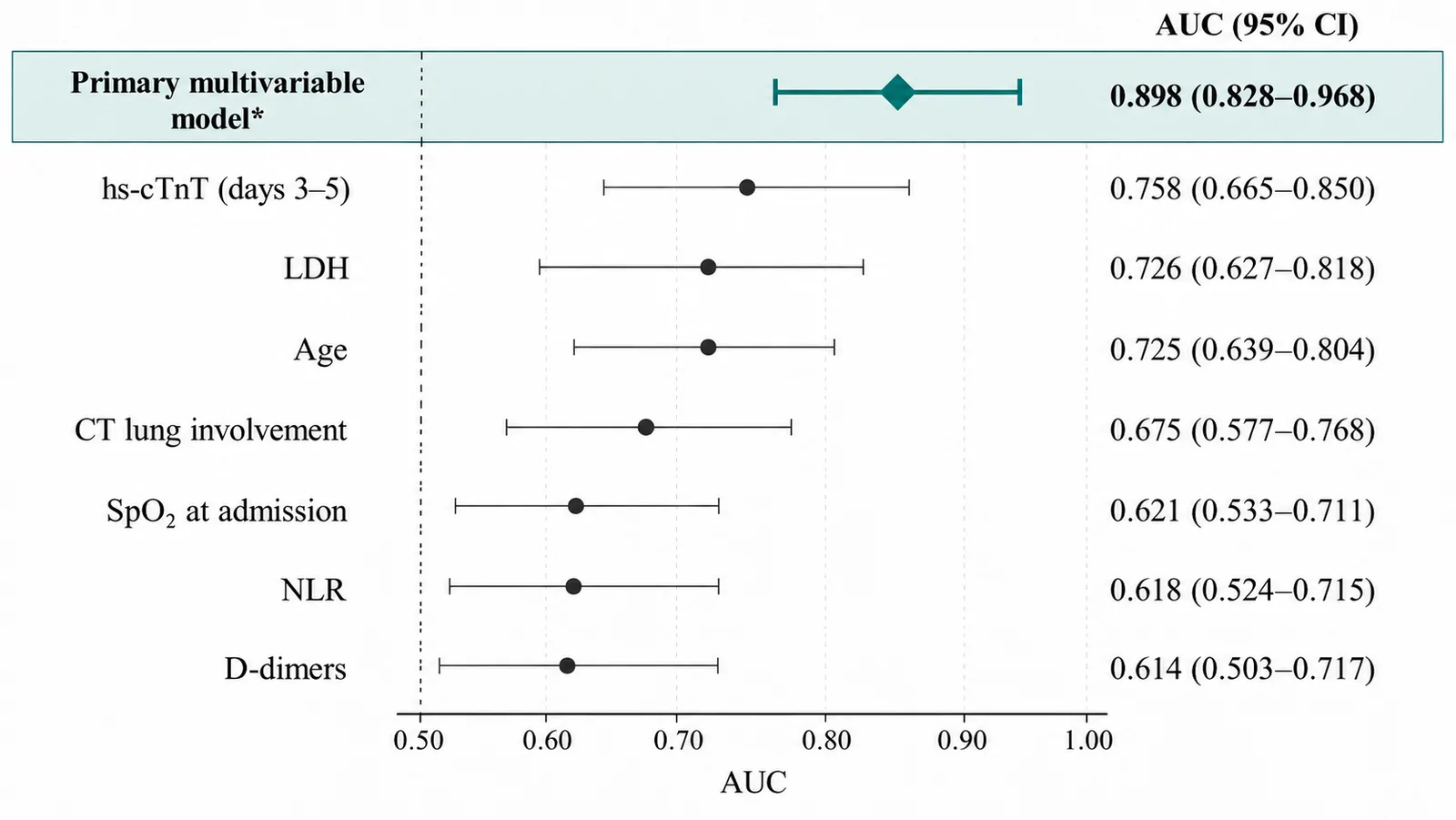
Discriminatory ability of individual prognostic variables and the primary multivariable model for in-hospital mortality. Points represent the AUC, and horizontal bars indicate 95% confidence intervals. The vertical dashed line represents an AUC of 0.50, corresponding to discrimination no better than chance. hs-cTnT was measured on days 3–5 of hospitalization; the remaining individual variables were assessed at admission. * The primary multivariable model included age, pre-existing cardiovascular disease, extent of pulmonary involvement on CT, and LDH. The multivariable model AUC (0.898) was calculated in the complete-case sample (N = 126; 37 deaths), whereas AUCs for individual predictors were calculated using the available observations for each variable and may therefore be based on differing sample sizes.

Among the individual variables evaluated, hs-cTnT measured on days 3–5 had an AUC of 0.758 (95% CI, 0.665–0.850). Among baseline predictors, LDH (AUC = 0.726; 95% CI, 0.627–0.818) and age (AUC = 0.725; 95% CI, 0.639–0.804) showed similar discrimination. The primary multivariable model achieved an apparent AUC of approximately 0.90 (Figure 1). ROC curves were not formally compared.

Given the observed prognostic association of hs-cTnT, the relationship between myocardial injury and in-hospital mortality was examined separately from the baseline prognostic model. Myocardial injury, defined as hs-cTnT > 14 ng/L, was present in 46 of the 138 patients with available hs-cTnT data. Overall, hs-cTnT was available in 138 of 172 patients. hs-cTnT availability varied across pandemic periods: 91.7% (44/48) in Pre-Alpha, 85.5% (65/76) in Alpha, 87.0% (20/23) in Delta, and 36.0% (9/25) in Omicron. Mortality was 29.0% (40/138) among patients with available hs-cTnT and 58.8% (20/34) among those without an available measurement. Accordingly, hs-cTnT was unavailable in 20/60 non-survivors (33.3%) compared with 14/112 survivors (12.5%). None of the 20 deaths among patients without an available hs-cTnT measurement occurred before day 5 after admission. Among the 46 patients with myocardial injury, 26 (56.5%) died during hospitalization, compared with 14 of 92 patients (15.2%) without myocardial injury. In unadjusted analysis, myocardial injury was associated with substantially higher odds of in-hospital mortality (OR = 7.24; 95% CI, 3.21–16.36; *p* < 0.001).

In the separate multivariable model, which included 121 patients with 35 deaths, myocardial injury remained significantly associated with in-hospital mortality after adjustment for age, pre-existing cardiovascular disease, and the extent of pulmonary involvement on CT (aOR = 5.19; 95% CI, 1.82–14.77; *p* = 0.002). Age also remained independently associated with mortality, with a 10-year increase associated with 1.56-fold higher odds of death (aOR = 1.56; 95% CI, 1.05–2.31; *p* = 0.028). Each 10% increase in the extent of pulmonary involvement on CT was associated with 1.46-fold higher odds of death (aOR = 1.46; 95% CI, 1.14–1.87; *p* = 0.003). In contrast, pre-existing cardiovascular disease was not independently associated with mortality in this model (aOR = 0.59; 95% CI, 0.19–1.80; *p* = 0.351). Using sex-specific 99th-percentile hs-cTnT thresholds, myocardial injury remained independently associated with mortality (aOR = 5.15; 95% CI, 1.80–14.73; *p* = 0.002).

In a secondary analysis integrating myocardial injury with severe respiratory involvement, 131 patients had complete data for classification into the four cardiorespiratory phenotypes, including 38 deaths (Supplementary Table S3). Mortality was 6.7% (2/30) among patients with neither myocardial injury nor respiratory involvement, 19.6% (11/56) among those with isolated respiratory involvement, 50.0% (3/6) among those with isolated myocardial injury, and 56.4% (22/39) among those with both myocardial and respiratory involvement. Because of sparse cells, the comparison between the combined myocardial and respiratory phenotype and the reference phenotype was reassessed using Firth penalized logistic regression (OR = 14.66; 95% CI, 4.04–79.46).

At hospital admission, 7 of 172 patients (4.1%) were classified as having critical COVID-19. Clinical indicators of disease severity during hospitalization were also strongly associated with in-hospital mortality (Table 4). Mortality was significantly higher among patients who met criteria for critical COVID-19 during hospitalization than among those who did not (73.8% vs. 1.1%; *p* < 0.001). As mechanical ventilation represented one of several criteria used to define critical disease, 60 of the 80 critically ill patients required ventilatory support. Similarly, mortality reached 91.7% among all patients who required mechanical ventilation, compared with 4.5% among those who did not (*p* < 0.001).

## 4. Discussion

In this single-center cohort of 172 patients hospitalized with severe or critical COVID-19, in-hospital mortality was 34.9%. Mortality was associated with three main domains: pre-existing vulnerability, reflected by age and cardiovascular disease; severity of acute illness, reflected by hypoxemia, pulmonary involvement, and LDH levels; and myocardial injury, indicated by elevated hs-cTnT. In the primary multivariable model, which included 126 patients with complete data, age, pre-existing cardiovascular disease, the extent of pulmonary involvement on CT and LDH were independently associated with in-hospital mortality. In a separate analysis, myocardial injury remained independently associated with mortality after adjustment for age, pre-existing cardiovascular disease, and pulmonary involvement. Together, these findings highlight the systemic nature of severe COVID-19 and the contribution of both pulmonary and extrapulmonary injury to prognosis.

The mortality observed in this study should be interpreted in the context of a population restricted to patients with severe or critical COVID-19 and recruited across several pandemic waves. Mortality differed significantly across epidemiological periods in our cohort. A meta-analysis comparing the Delta and Omicron periods reported better outcomes during the Omicron period at the population level, although substantial mortality persisted among patients who developed critical illness [13]. Differences between pandemic waves may reflect several factors, including the predominant viral variant, vaccination and post-infection immunity, changes in evidence-based treatment, availability of antiviral and immunomodulatory therapies, admission criteria, and pressure on healthcare systems. The mortality rate of 34.9% observed in our cohort is consistent with rates reported in other high-risk cohorts of critically ill or mechanically ventilated patients with COVID-19 [8]. However, it reflects a selected high-risk hospitalized population and should not be extrapolated to all individuals with SARS-CoV-2 infection or to all hospitalized patients with COVID-19. Despite these temporal differences, older age and LDH remained associated with in-hospital mortality after additional adjustment for pandemic period, whereas the association with pre-existing cardiovascular disease was attenuated. However, the period-adjusted model should be interpreted cautiously because of the limited number of deaths and sparse event counts across pandemic periods.

Age showed a consistent association with mortality and remained significant after adjustment. This finding is consistent with previous studies linking older age to poorer outcomes in COVID-19, potentially through greater frailty, immunosenescence, reduced cardiopulmonary reserve, and a higher burden of multimorbidity [14]. Cardiovascular and chronic kidney disease were more common among non-survivors, and cardiovascular disease remained independently associated with mortality in the primary model. However, the association of pre-existing cardiovascular disease was not stable across secondary models, suggesting model- and sample-related instability rather than a protective association. This is consistent with meta-analyses showing an association between pre-existing cardiovascular disease and severe COVID-19 and mortality [5,14].

Overall comorbidity burden, assessed using the ACCI, was also higher among non-survivors (median 4 [IQR, 3–6] vs. 3 [IQR, 1–4]; *p* < 0.001). Because age is incorporated into the ACCI, this finding is not independent of the observed association between age and mortality. Rather, the ACCI provides an integrated measure of age and comorbidity burden that may complement initial clinical risk assessment. However, ACCI/CCI thresholds derived from other cohorts should not be directly applied without appropriate validation.

Among the laboratory markers evaluated, LDH remained strongly associated with mortality after adjustment in the primary model. LDH and age also had similar discriminatory ability, with AUCs of 0.726 and 0.725, respectively. While age reflects biological vulnerability and comorbidity burden, elevated LDH may reflect the extent of tissue injury and systemic disease severity. Both remained independently associated with mortality after adjustment, supporting their prognostic relevance in this cohort. Previous meta-analyses have similarly reported associations between LDH levels, NLR, D-dimer levels, and adverse COVID-19 outcomes, although with substantial heterogeneity across populations and laboratory methods [15].

In our cohort, NLR and D-dimer levels were higher among non-survivors, whereas CRP, platelet count, creatinine, and ferritin at admission did not differ significantly between groups. Post-baseline laboratory findings showed a different pattern. hs-cTnT, measured according to the study protocol on days 3–5, was significantly higher among non-survivors, whereas hs-CRP measured at the same time point was numerically higher among non-survivors (median 26.1 [IQR, 6.98–92.7] vs. 11.6 [IQR, 2.51–43.1]) but did not reach statistical significance (*p* = 0.069). Maximum CK during hospitalization was also higher among non-survivors. These findings illustrate differences in laboratory profiles both at baseline and during hospitalization; however, post-baseline measurements should be interpreted as markers obtained during the clinical course rather than as predictors available at admission.

Among the individual variables evaluated, hs-cTnT measured on days 3–5 showed the highest discriminatory ability. Myocardial injury, defined as hs-cTnT > 14 ng/L, identified a subgroup with markedly higher mortality and remained independently associated with death after adjustment for age, pre-existing cardiovascular disease, and the extent of pulmonary involvement on CT. Because hs-cTnT was measured after admission, this finding should be interpreted as prognostic information obtained during hospitalization rather than as a baseline predictor. This finding is consistent with meta-analyses showing an association between elevated cardiac troponin and mortality in COVID-19 after adjustment for major clinical risk factors [16]. However, hs-cTnT elevation is not etiologically specific and indicates myocardial injury without establishing its underlying cause. Potential mechanisms include oxygen supply–demand mismatch, systemic inflammation, microthrombosis, renal dysfunction, myocardial infarction, and, less commonly, myocarditis [17]. Without additional diagnostic data, elevated hs-cTnT should not be interpreted as evidence of direct viral myocardial injury.

The extent of pulmonary involvement on CT and oxygen saturation at admission reflected the initial severity of respiratory disease. Greater CT involvement was associated with mortality in unadjusted analysis, while its prognostic contribution varied across adjusted analyses: it remained independently associated with mortality in the primary and myocardial injury models, although with borderline statistical significance in the primary model, but not after adjustment for pandemic period. Previous meta-analytic evidence has similarly linked greater CT severity to mortality, while also showing substantial variation in scoring methods and thresholds [18]. Consistent findings were reported by Hălmaciu et al. in a single-center observational study, in which greater CT-assessed pulmonary involvement was associated with adverse outcomes [19]. In this cohort, CT findings provided complementary information on pulmonary disease burden alongside clinical, oxygenation, and extrapulmonary parameters.

Mortality was particularly high among patients with combined myocardial and respiratory involvement, although estimates across cardiorespiratory phenotypes should be interpreted cautiously because of the small subgroup size. This finding supports an integrated assessment of cardiac and pulmonary involvement in severe COVID-19, as hypoxemia, extensive pulmonary injury, systemic inflammation, endothelial dysfunction, and hemodynamic stress may all contribute to myocardial injury. Similar findings were reported by Kavosi et al. [20], who identified both myocardial injury (OR = 2.19; 95% CI, 1.14–4.18) and severe pulmonary involvement on CT (OR = 2.24; 95% CI, 1.15–4.34) as independent predictors of mortality in a cohort of 389 patients with COVID-19. Importantly, this analysis combined respiratory involvement assessed at admission with myocardial injury assessed on days 3–5 and should therefore be interpreted as an integrated assessment across the early clinical course rather than as a purely baseline phenotype. However, our findings demonstrate an association between the cardiorespiratory phenotype and mortality but do not establish causality; a formal statistical interaction between cardiac and pulmonary injury was not assessed.

CT-assessed pulmonary involvement showed modest discriminatory ability (AUC = 0.675; 95% CI, 0.577–0.768). Oxygen saturation at admission, NLR, and D-dimer levels showed AUC values of approximately 0.60, indicating limited discrimination when used individually. These findings suggest that the prognostic value of individual markers may be greater when considered alongside other clinical and laboratory characteristics rather than in isolation.

The multivariable model including age, pre-existing cardiovascular disease, extent of pulmonary involvement on CT, and LDH showed an apparent AUC of 0.898 (95% CI, 0.828–0.968), indicating good discrimination in the study cohort. For LDH, multivariable adjustment revealed a stronger association than the unadjusted estimate, consistent with negative confounding (suppression); however, given the sample size and the relatively wide confidence interval, the magnitude of the adjusted estimate should be interpreted cautiously. Its higher AUC compared with those of the individual predictors suggests that combining information on patient vulnerability, cardiovascular comorbidity, pulmonary involvement, and tissue injury may improve risk assessment. However, because the ROC curves were not formally compared, the model cannot be considered statistically superior to the individual predictors based on these data alone.

The model’s performance should be interpreted cautiously because the AUC was estimated in the same cohort used to develop the model. The complete-case analysis included 126 patients and 37 deaths, and the relatively small number of events and exclusion of patients with missing data may have resulted in optimistic performance estimates. Internal bootstrap validation showed limited optimism and preserved good discrimination; however, external validation in an independent cohort is required before considering the model for clinical application.

The present study provides an integrated assessment of demographic, cardiovascular, imaging, and biological factors associated with in-hospital mortality in a real-world cohort of adults with severe or critical COVID-19 across multiple pandemic waves. The primary model identified age, pre-existing cardiovascular disease, the extent of pulmonary involvement on CT and LDH as independent factors associated with mortality using information available at or shortly after admission. Separately, myocardial injury identified by elevated hs-cTnT on days 3–5 remained independently associated with mortality and, when considered together with severe respiratory involvement, identified a subgroup with particularly high mortality. These findings support a multidimensional approach to prognostic assessment that incorporates both baseline risk and information emerging during the early in-hospital course. The relevance of these findings lies in demonstrating that prognostic assessment in a clinically homogeneous, high-risk population may draw on complementary information obtained at different stages of hospitalization: age, cardiovascular disease, and LDH for initial risk assessment, and myocardial injury identified on days 3–5 for subsequent prognostic evaluation. The cardiorespiratory analysis further suggests that combining information on severe respiratory involvement and myocardial injury may help identify a particularly high-risk phenotype.

## 5. Strengths and Limitations

This study has several strengths, including detailed clinical, laboratory, imaging, and treatment data from a well-defined cohort of patients with severe or critical COVID-19, the use of in-hospital mortality as an objective outcome, and the combined assessment of cardiac and respiratory involvement. In addition, the primary prognostic model focused on variables available at or shortly after admission and excluded subsequent markers of clinical deterioration.

Several limitations should also be acknowledged. The single-center design and relatively small sample size limit the generalizability of the findings and leave the possibility of residual confounding. The number of deaths and the use of complete-case analysis limited model complexity and may have introduced bias related to missing data. Notably, patients excluded from the complete-case analysis had higher in-hospital mortality than those included, indicating potential selection bias. Although multiple imputation sensitivity analysis yielded broadly consistent estimates for age and LDH, the associations with pulmonary involvement on CT, and pre-existing cardiovascular disease were less robust and should therefore be interpreted cautiously. BMI was available in only 77 of 172 patients and was therefore not included in the prognostic models. hs-cTnT was measured on days 3–5 rather than at admission; among patients without an available hs-cTnT measurement, no deaths occurred before day 5, reducing the likelihood that missing hs-cTnT measurements were primarily attributable to very early mortality. However, because hs-cTnT missingness was more frequent among non-survivors, non-random missingness and availability-related selection bias cannot be excluded. Therefore, the myocardial injury analysis reflects prognostic information obtained during hospitalization and should not be interpreted as baseline risk prediction. Systematic echocardiographic assessment was not available, limiting further characterization of the mechanisms underlying myocardial injury. The study also covered several pandemic waves, during which circulating variants, population immunity, treatment strategies, and access to intensive care changed over time. Furthermore, although bootstrap internal validation showed limited optimism and acceptable calibration, external validation in an independent cohort was not performed, limiting the generalizability of the model and its applicability to individual risk prediction. Multiple analyses and small subgroups also increase the possibility of chance findings, particularly in exploratory analyses.

### Clinical Implications

This study supports the use of a multimodal approach to risk assessment in severe or critical COVID-19. Our findings show that baseline age, cardiovascular disease, and LDH help characterize initial risk, while detection of myocardial injury during the first days of hospitalization may identify patients with an unfavorable clinical course. However, these findings should not be used to derive a treatment algorithm without further validation.

Future studies should prospectively validate these findings in independent cohorts and assess both model discrimination and calibration. Serial measurements of LDH and hs-cTnT may provide additional information on disease progression, while more detailed cardiac assessment could help clarify the mechanisms and prognostic significance of myocardial injury.

## 6. Conclusions

In this cohort of adults with severe or critical COVID-19, in-hospital mortality was associated with demographic, cardiovascular, respiratory, and laboratory factors, highlighting the multifactorial nature of prognosis. Age, pre-existing cardiovascular disease, extent of pulmonary involvement on CT and LDH were independently associated with mortality in the primary multivariable model; however, the cardiovascular disease and pulmonary involvement associations were attenuated in the multiple-imputation sensitivity analysis. Myocardial injury, defined by elevated hs-cTnT measured on days 3–5 of hospitalization, also remained independently associated with mortality after adjustment for age, cardiovascular disease, and the extent of pulmonary involvement.

The primary multivariable model, which integrated clinical, imaging, and laboratory parameters, showed good apparent discriminatory ability, while patients with both myocardial and respiratory involvement had particularly high mortality. These findings support a multidimensional approach to risk assessment in severe or critical COVID-19 but require validation in independent cohorts before they can be used to develop clinical prediction tools.

## Figures and Tables

**Table 1:** Demographic, clinical, and laboratory characteristics according to in-hospital survival.

Variable	Total	Survivors, n = 112	Non-Survivors, n = 60	*p* Value
Age, years	67.0 (54.0–77.0)	63.0 (53.0–73.2)	75.5 (66.0–82.2)	<0.001
Male sex, n/N (%)	106/172 (61.6)	74/112 (66.1)	32/60 (53.3)	0.138
Time from symptom onset to hospital admission, days	6 (4–8)	7 (4–9)	6 (4–7)	0.237
Hypertension, n/N (%)	99/170 (58.2)	59/112 (52.7)	40/58 (69.0)	0.049
Cardiovascular disease, n/N (%)	54/170 (31.8)	26/112 (23.2)	28/58 (48.3)	0.002
Diabetes mellitus, n/N (%)	35/170 (20.6)	22/112 (19.6)	13/58 (22.4)	0.692
COPD, n/N (%)	11/170 (6.5)	5/112 (4.5)	6/58 (10.3)	0.188
Chronic kidney disease, n/N (%)	18/170 (10.6)	6/112 (5.4)	12/58 (20.7)	0.003
Malignancy, n/N (%)	11/170 (6.5)	6/112 (5.4)	5/58 (8.6)	0.513
Cerebrovascular disease, n/N (%)	6/170 (3.5)	3/112 (2.7)	3/58 (5.2)	0.411
SpO_2_ at admission, %	89.0 (85.0–91.0)	89.0 (86.0–92.0)	87.0 (82.0–89.5)	0.009
CT lung involvement, %	40.0 (25.0–56.3)	35.0 (25.0–50.0)	50.0 (35.0–65.0)	<0.001
CRP at admission, mg/L	80.7 (46.2–154.6)	80.7 (47.9–148.3)	78.3 (40.0–185.9)	0.852
NLR	6.9 (3.8–11.0)	6.6 (3.1–9.8)	8.4 (5.2–14.4)	0.022
Platelet count, /µL	195,700 (149,000–250,000)	194,350 (143,500–240,000)	205,000 (158,350–280,000)	0.286
LDH at admission, U/L	341.5 (274.2–470.0)	321.0 (250.8–408.2)	497.0 (314.2–730.8)	<0.001
Creatinine at admission, mg/dL	0.9 (0.8–1.2)	0.89 (0.77–1.1)	1.1 (0.76–1.43)	0.178
Ferritin at admission, ng/mL	825.2 (503.9–1385.6)	783.7 (476.7–1278.2)	939.4 (628.6–1916.6)	0.091
hs-cTnT, ng/L	9.2 (5.8–15.8)	7.3 (5.4–13.1)	20.1 (10.0–28.2)	<0.001
hs-CRP, mg/L	16.4 (3.46–49.7)	11.6 (2.51–43.1)	26.1 (6.98–92.7)	0.069
D-dimer, mg/L	0.9 (0.4–2.3)	0.7 (0.4–1.6)	1.6 (0.5–3.8)	0.031
Maximum CK, U/L	147.5 (64.75–358.75)	113.5 (55–256)	295.5 (110–637)	<0.001
AST at admission, U/L	42 (30.75–64.25)	38 (25–76)	55.5 (38.5–76.3)	0.002
ALT at admission, U/L	39.5 (26.0–69.75)	40 (28–69)	38 (22–70)	0.819
Critical COVID-19 during hospitalization, n/N (%)	80/172 (46.5)	21/112 (18.8)	59/60 (98.3)	<0.001
ICU transfer, n/N (%)	80/172 (46.5)	21/112 (18.8)	59/60 (98.3)	<0.001
COVID-19 vaccination, n/N (%)	5/172 (2.9)	1/112 (0.9)	4/60 (6.7)	0.051

Note: Data are presented as median [interquartile range, IQR] for continuous variables and n/N (%) for categorical variables. Unless otherwise specified, laboratory parameters represent values obtained within the first 24 h of hospital admission. hs-CRP and hs-cTnT were measured from the same blood sample collected on days 3–5 after admission, according to the study protocol. CK max represents the highest creatine kinase level recorded during hospitalization. *p* values refer to comparisons between survivors and non-survivors. Critical COVID-19 during hospitalization and ICU transfer each occurred in 80 patients but did not involve identical patient groups: 74 patients had both, 6 had critical COVID-19 without ICU transfer, and 6 had ICU transfer without recorded critical COVID-19 status.

**Table 2:** Factors associated with in-hospital mortality: univariable logistic regression analysis.

Predictor	Crude OR	95% CI	*p* Value
Age, per 10-year increase	1.86	1.42–2.43	<0.001
Male sex	0.59	0.31–1.11	0.103
Hypertension	2.00	1.02–3.90	0.043
Cardiovascular disease	3.09	1.57–6.07	0.001
Diabetes mellitus	1.18	0.55–2.56	0.672
Malignancy	1.67	0.49–5.71	0.416
Cerebrovascular disease	1.98	0.39–10.14	0.412
COPD	2.47	0.72–8.47	0.150
Chronic kidney disease	4.61	1.63–13.03	0.004
SpO_2_, per 5% decrease	1.41	1.08–1.83	0.010
CT lung involvement, per 10% increase	1.33	1.12–1.58	0.001
CRP, per doubling	0.97	0.79–1.18	0.736
NLR, per doubling	1.33	1.01–1.77	0.045
Platelets, per 50,000/µL decrease	0.86	0.72–1.02	0.087
LDH, per doubling	3.99	2.14–7.42	<0.001
Creatinine, per doubling	1.51	0.91–2.50	0.112
Ferritin, per doubling	1.29	0.95–1.74	0.102
hs-cTnT, per doubling	2.13	1.50–3.03	<0.001
D-dimers, per doubling	1.23	1.02–1.48	0.028

Note: Continuous biomarkers with skewed distributions (CRP, NLR, LDH, creatinine, ferritin, hs-cTnT, and D-dimers) were log2-transformed; their ORs therefore represent the change in odds associated with a doubling of the biomarker value.

**Table 3:** Factors independently associated with in-hospital mortality: multivariable logistic regression analysis.

Predictor	aOR	95% CI	*p* Value
Age, per 10-year increase	2.55	1.55–4.22	<0.001
Cardiovascular disease	3.06	1.03–9.07	0.044
CT lung involvement, per 10% increase	1.34	1.01–1.80	0.046
LDH level, per doubling	6.31	2.70–14.78	<0.001

**Table 4:** Clinical course and in-hospital mortality.

Clinical Course	N	Survivors, n = 112	Non-Survivors, n = 60	Mortality	*p* Value
Critical COVID-19					
No	92	91	1	1.1%	<0.001
Yes	80	21	59	73.8%	
Need for mechanical ventilation					
No	112	107	5	4.5%	<0.001
Yes	60	5	55	91.7%	

## Data Availability

Data supporting the results of this article will be provided on request, by the corresponding author.

## Supplementary Materials

The following supporting information can be downloaded at: https://www.mdpi.com/article/10.3390/medicina62091775/s1, Figure S1: Flow of the study and analytic samples; Table S1: Treatment patterns according to pandemic period and in-hospital outcome; Table S2: Sensitivity analysis of the primary multivariable mortality model using multiple imputation; Table S3: In-hospital mortality according to cardiorespiratory phenotype.
