# Supplementary material for: Myocardial Injury and Predictors of In-Hospital Mortality in Severe or Critical COVID-19: A Single-Center Observational Cohort Study
Source: Medicina (Kaunas). 2026 Sep 16;62(9):1775. doi: 10.3390/medicina62091775 (PMC13608992; doi:10.3390/medicina62091775)
Supplement: Supplementary file 1 [file medicina-62-01775-s001.zip › Supplementary Tables S1-S3.pdf]

Table S1. Treatment patterns according to pandemic period and in-hospital outcome

Treatment	Total (N=172)	Pre-Alpha (n=48)	Alpha (n=76)	Delta (n=23)	Omicron (n=25)	Survivors (n=112)	Non-survivors (n=60)
Remdesivir, n (%)	100 (58.1)	28 (58.3)	43 (56.6)	15 (65.2)	14 (56.0)	62 (55.4)	38 (63.3)
Favipiravir, n (%)	61 (35.5)	16 (33.3)	27 (35.5)	12 (52.2)	6 (24.0)	46 (41.1)	15 (25.0)
Tocilizumab, n (%)	24 (14.0)	13 (27.1)	9 (11.8)	1 (4.3)	1 (4.0)	15 (13.4)	9 (15.0)
Anakinra, n (%)	13 (7.6)	0 (0.0)	0 (0.0)	4 (17.4)	9 (36.0)	5 (4.5)	8 (13.3)
Anticoagulation, n (%)	168 (97.7)	47 (97.9)	74 (97.4)	23 (100.0)	24 (96.0)	110 (98.2)	58 (96.7)
Corticosteroids, n (%)	171 (99.4)	48 (100.0)	76 (100.0)	23 (100.0)	24 (96.0)	111 (99.1)	60 (100.0)
Antibiotics, n (%)	167 (97.1)	46 (95.8)	74 (97.4)	23 (100.0)	24 (96.0)	108 (96.4)	59 (98.3)

Note: Data are presented as n (%).

Table S2. Sensitivity analysis of the primary multivariable mortality model using multiple imputation

Predictor	Pooled OR	95 % CI	p-value
Age, per 10-year increase	2.30	1.58-3.33	<0.001
Pre-existing cardiovascular disease	1.92	0.79-4.69	0.150
CT involvement, per 10% increase	1.26	1.00-1.59	0.054
LDH, per doubling	5.03	2.38-10.61	<0.001

Note: Missing data were handled using multiple imputation by chained equations with 50 imputations; estimates were pooled using Rubin's rules. OR, odds ratio; CI, confidence interval; CT, computed tomography; LDH, lactate dehydrogenase.

Table S3. In-hospital mortality according to cardiorespiratory phenotype

Cardiorespiratory phenotype	Deaths/N	Mortality, %
Neither myocardial injury nor severe respiratory involvement	2/30	6.7
Isolated severe respiratory involvement	11/56	19.6
Isolated myocardial injury	3/6	50.0
Combined myocardial and severe respiratory involvement	22/39	56.4

Note: The analytic sample included 131 patients, including 38 deaths. Because of sparse cells, the comparison between the combined myocardial and respiratory phenotype and the reference phenotype (neither myocardial injury nor severe respiratory involvement) was reassessed using Firth penalized logistic regression (OR = 14.66; 95% CI, 4.04–79.46).
